# Validation and assessment of variant calling pipelines for next-generation sequencing

**DOI:** 10.1186/1479-7364-8-14

**Published:** 2014-07-30

**Authors:** Mehdi Pirooznia, Melissa Kramer, Jennifer Parla, Fernando S Goes, James B Potash, W Richard McCombie, Peter P Zandi

**Affiliations:** 1 Department of Psychiatry and Behavioral Sciences, Johns Hopkins University, Baltimore, MD 21205, USA; 2 Stanley Institute for Cognitive Genomics, Cold Spring Harbor Laboratory, Woodbury, NY 11797, USA; 3 Department of Psychiatry, Carver College of Medicine, University of Iowa School of Medicine, Iowa City, IA 52242, USA; 4 Watson School of Biological Science, Cold Spring Harbor Laboratory, Cold Spring Harbor, NY 11724, USA; 5 Department of Mental Health, Johns Hopkins Bloomberg School of Public Health, Baltimore, MD 21205, USA

**Keywords:** Variant calling pipelines, Next-generation sequencing, Exome sequencing

## Abstract

**Background:**

The processing and analysis of the large scale data generated by next-generation sequencing (NGS) experiments is challenging and is a burgeoning area of new methods development. Several new bioinformatics tools have been developed for calling sequence variants from NGS data. Here, we validate the variant calling of these tools and compare their relative accuracy to determine which data processing pipeline is optimal.

**Results:**

We developed a unified pipeline for processing NGS data that encompasses four modules: mapping, filtering, realignment and recalibration, and variant calling. We processed 130 subjects from an ongoing whole exome sequencing study through this pipeline. To evaluate the accuracy of each module, we conducted a series of comparisons between the single nucleotide variant (SNV) calls from the NGS data and either gold-standard Sanger sequencing on a total of 700 variants or array genotyping data on a total of 9,935 single-nucleotide polymorphisms. A head to head comparison showed that Genome Analysis Toolkit (GATK) provided more accurate calls than SAMtools (positive predictive value of 92.55% vs. 80.35%, respectively). Realignment of mapped reads and recalibration of base quality scores before SNV calling proved to be crucial to accurate variant calling. GATK HaplotypeCaller algorithm for variant calling outperformed the UnifiedGenotype algorithm. We also showed a relationship between mapping quality, read depth and allele balance, and SNV call accuracy. However, if best practices are used in data processing, then additional filtering based on these metrics provides little gains and accuracies of >99% are achievable.

**Conclusions:**

Our findings will help to determine the best approach for processing NGS data to confidently call variants for downstream analyses. To enable others to implement and replicate our results, all of our codes are freely available at http://metamoodics.org/wes.

## Background

Advances in next-generation sequencing (NGS) technology are beginning to provide a cost-effective approach for identifying and cataloging the full spectrum of genetic variation across the genome at a scale not previously attainable by more traditional techniques such as Sanger sequencing or single-nucleotide polymorphism (SNP) arrays, thus creating a foundation for a profound understanding of human diseases [1-4]. The ability to comprehensively examine the genome in a high-throughput and unbiased manner has generated a great deal of interest in the use of NGS platforms to sequence entire exome or genome of large numbers of individuals to search variation in common disease, mutations underlying rare Mendelian disease [5,6], or spontaneously arising variation for which no gene-mapping shortcuts are available (e.g., somatic mutations in cancer [7,8] or *de novo* mutations in autism [9-13] and schizophrenia [14]).

Although NGS is a powerful approach, there are many technical challenges involved in obtaining a complete and accurate record of sequence variation from NGS data and in turning raw sequence reads into biologically meaningful information [15-17]. Given accurately mapped and calibrated reads, identifying simple SNPs, let alone more complex variation such as multiple base pair substitutions, insertions, deletions, inversions, and copy number variation, requires complex statistical models and sophisticated bioinformatics tools to implement these models on large amounts of data [16,18]. A number of such tools have recently been developed, including the short oligonucleotide alignment program (SOAP) [19,20], SAMtools [21], and the Genome Analysis Toolkit (GATK) [22]. However, many questions remain about how well these different tools work in identifying and accurately calling sequence variation and what are the best strategies for optimizing their use. Several recent studies have begun to evaluate and compare the performance of these tools [23-25].

We sought to add to these studies in order to determine best processes for identifying and calling sequence variants from NGS data. We carried out a comparative analysis of 130 whole exome subjects from an ongoing bipolar disorder exome sequencing project. We developed a multi-stage pipeline for processing the exome data on these subjects and then examined the accuracy of calls derived from different implementations of the pipeline by validation with Sanger sequencing of a total of 700 variants using the ABI capillary sequencing platform and SNP genotyping on a total of 9,935 variants using the Affymetrix microarray platform. The goal was to critically evaluate and optimize processes for generating valid single nucleotide variant (SNV) calls from NGS data. Our results provide useful information and guidance for future studies analyzing data from next-generation sequencing experiments.

## Results and discussion

### Pipeline development

**Figure 1:**
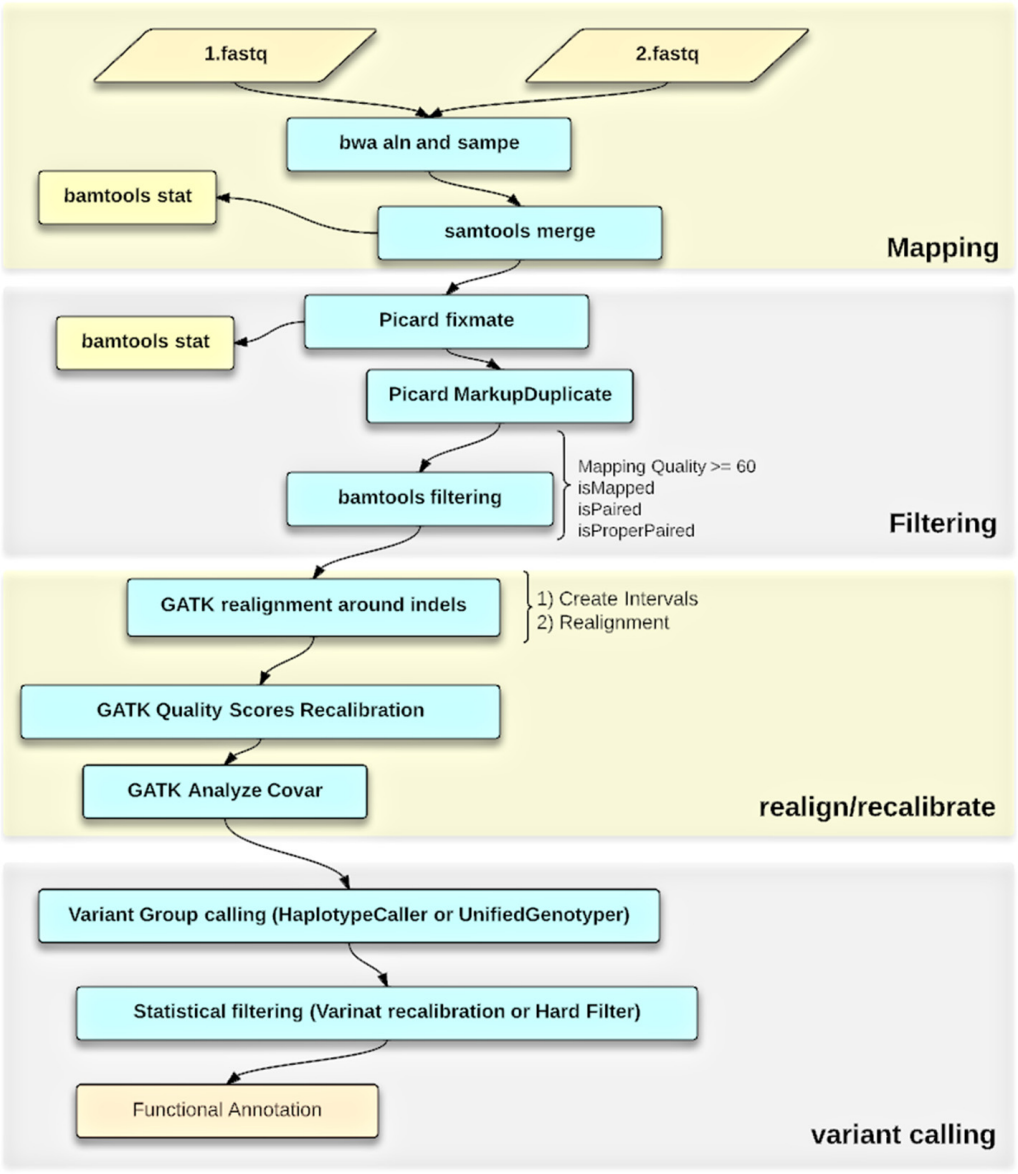
**Modular structure of pipeline for processing next-generation sequencing data.** The pipeline contains 4 modules: (1) mapping, (2) filtering, (3) realignment/recalibration, and (4) variant calling. Detailed description is available at http://metamoodics.org/wes.

We developed a modular pipeline for processing NGS as shown in Figure 1 and described in Additional file 1 and in more detail at our Wiki site (http://metamoodics.org/wes). First, raw read data with well-calibrated base error estimates in fastq format are mapped to the reference genome. The BWA mapping (version 0.7.0) application [26] is used to map reads to the human genome reference, allowing for two mismatches in 30-base seeds, and generate a technology-independent SAM/BAM reference file format [21]. Next, duplicate fragments are marked and eliminated with Picard (version 1.8) (http://picard.sourceforge.net), mapping quality is assessed and low-quality mapped reads are filtered, and paired read information is evaluated to ensure that all mate-pair information is in sync between each read. We then refine the initial alignments by local realignment and identify suspicious regions. Using this information as a covariate along with other technical covariates and known sites of variation, the GATK base quality score recalibration (BQSR) is carried out. Lastly, SNV calling is performed using the recalibrated and realigned BAM files.

In this study, we evaluated different components of the pipeline that may influence the accuracy of the SNV calls in order to optimize the pipeline. We did this by comparing SNV call sets from the pipeline versus ‘gold standard’ calls either from targeted Sanger sequencing or previously available genome-wide association study (GWAS) data. In particular, we compared two of the most commonly used tools for variant calling (SAMtools versus GATK), different algorithms for variant calling implemented by GATK (UnifiedGenotyper versus HaplotypeCaller and hard filtering versus VariantRecalibration), and the influence of several sequence parameters (read depth, allele balance, and mapping quality).

### GATK versus SAMtools

**Figure 2:**
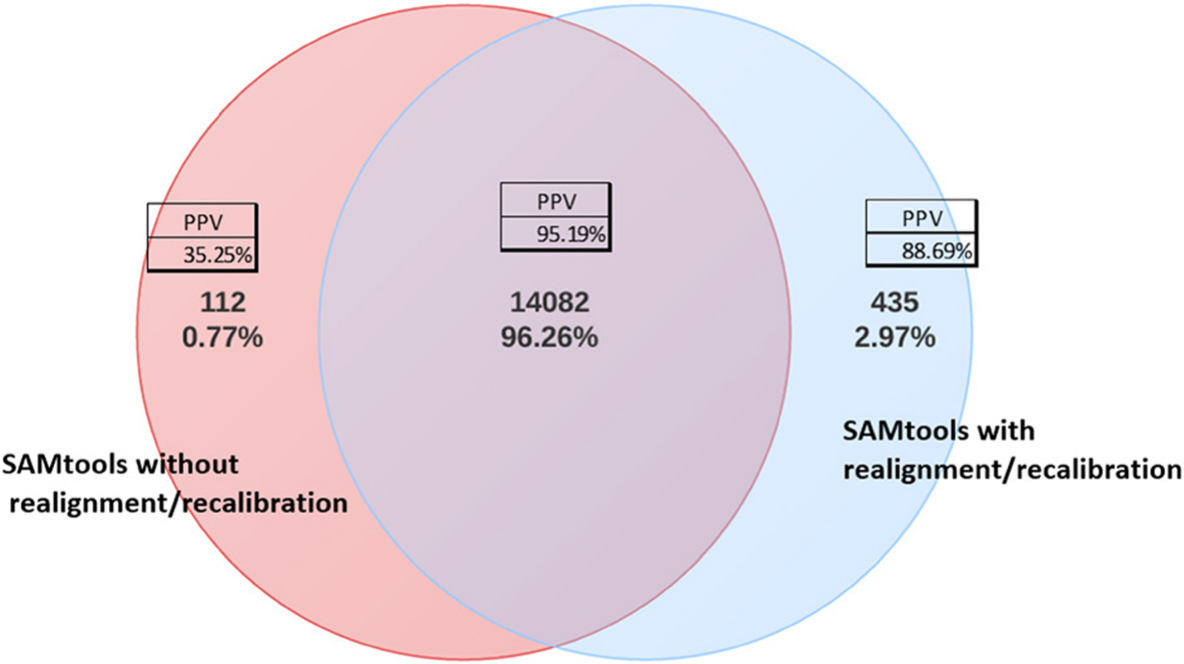
**Comparison of SNV calling using SAMtools with and without realignment/recalibration on a sample of 30 subjects.** Sanger sequencing was performed to evaluate the accuracy of these calls.

A number of tools have been developed for variant calling from aligned sequence reads, including GATK [22], SAMtools [21], MAQ [27], VarScan [28], SNVer [29], GNUMAP [30], and SOAPsnp [31]. We sought to compare GATK (version 2.6) and SAMtools (version 0.1.18), which are among the most widely used. Before making this comparison, we first evaluated the effect of realignment and recalibration of sequences on the accuracy of downstream variant calling. We did this by comparing SNV call sets from SAMtools with and without realignment/recalibration on a sample of 30 subjects with an average of 14,730 SNVs per subject. As shown in Figure 2, the majority of SNVs, approximately 96% of all SNVs called by either of the call sets, were called by both. Less than 1% of all SNVs were called only by the pipeline that did not use realignment/recalibration, while another 3% of all SNVs were called only by the pipeline with realignment/recalibration. We resequenced with Sanger methods a random selection of identified variants to evaluate the accuracy of these calls. A total of 341 individual SNV calls were available to evaluate the pipeline with realignment/recalibration, for which we observed a positive predictive value of 88.69% among variants that were called only after realignment/recalibration. By contrast, we found a positive predictive value of only 35.25% among individual SNV calls for the pipeline without realignment/recalibration only. Similar to others [23,32], we concluded based on these findings that realignment/recalibration improves the accuracy of calls and implemented these steps in our pipeline as standard practice moving forward.

**Figure 3:**
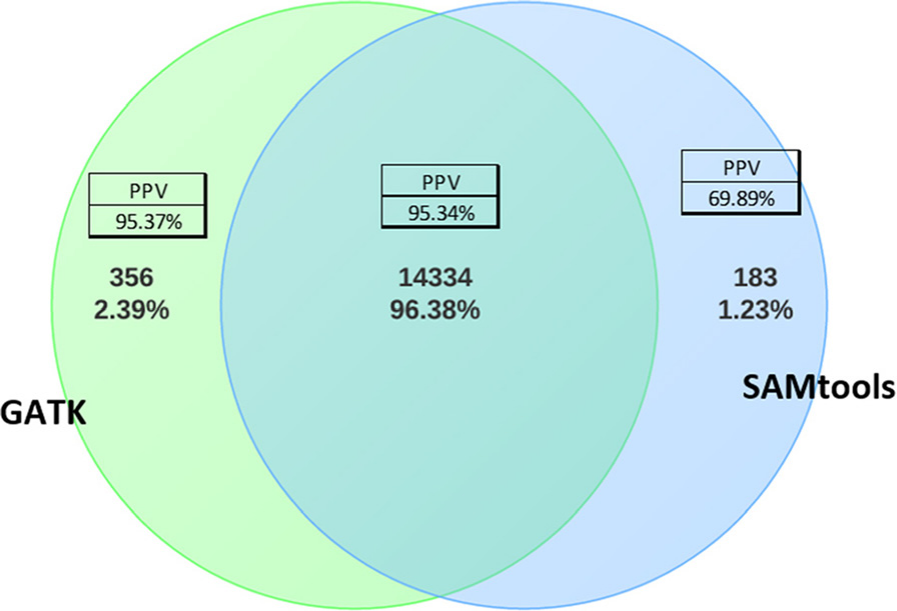
**Comparison of SNVs calls from GATK versus SAMtools using data from 30 subjects.** For these comparisons, we used the UnifiedGenotyper algorithm in GATK and mpileup in SAMtools. Sanger sequencing was performed to evaluate the accuracy of these calls.

We then compared SNV calls from GATK versus SAMtools using data from the same 30 subjects (Figure 3). For these comparisons, we used the UnifiedGenotyper algorithm in GATK and mpileup in SAMtools. We resequenced 336 individual calls from GATK and observed a true-positive rate of 95.00%. By contrast, from calls only made by SAMtools (1.23% of the total calls), we resequenced 341 individual calls and observed a much lower true-positive rate of 69.89%. We considered whether it would be better to make calls using both tools and take the intersection as the final call set. Just over 96.38% of all SNVs called by either tool were called by both. We resequenced 165 individual calls of these SNVs and observed a positive predictive value of 95.34%. Another 2.39% of all SNVs were called only by GATK. Resequencing of 171 individual calls of these variants yielded a positive predictive value of 95.37%. As a result, we decided to go with GATK exclusively as our variant calling tool. Additional file 2: Table S1 provides a breakdown of the characteristics of the SNV calls that were concordant and discordant with the Sanger sequencing by the different calling methods.

### Variant quality score recalibration versus hard filter

Moving forward with GATK, we examined the accuracy of calls when using hard filtering with recommended thresholds from GATK (variant confidence score ≥30, mapping quality ≥40, read depth ≥6, and strand bias FSfilter <60); a full description is provided in Additional file 1 versus using GATK's Variant Quality Score Recalibration (VQSR), which builds a Gaussian mixture model by looking at the annotation values over a high-quality subset of the input call set and then uses this model to evaluate all input variants. We compared calls using both strategies against GWAS SNP genotype data previously obtained from 100 subjects and 9,930 SNVs. We used the UnifiedGenotyper algorithm for these comparisons. A total of 181,304 out of 191,361 (94.74%) total SNVs were called in common between the hard filtering and VQSR strategies. Table 1 shows a breakdown of genotypes for these 181,304 SNVs. Over 99% of individual genotype calls at the SNVs were concordant between both strategies. As a result, the sensitivity and specificity of VQSR versus hard filtering using the GWAS SNP genotype as the gold standard were very similar, with sensitivity of 99.87% for both VQSR and hard filtering, and specificity of 99.79% and 99.56% for VQSR and hard filtering, respectively. In order to evaluate the differences more closely, we examined the small percentage of discordant genotype calls between VQSR and hard filtering. Here, the calls from VQSR were almost always in better agreement with the available GWAS SNP genotype data than were the calls from hard filtering (1,227 out of 1,233 calls in agreement for VQSR vs. 6 out of 1,233 for hard filtering). To evaluate the differences with respect to rarer SNVs with minor allele frequency (MAF) <10% that are not available in the GWAS data, we randomly selected 50 rarer SNVs from the subset that were discordantly called between VQSR and hard filtering and performed Sanger sequencing to validate the calls. Again, the VQSR calls were in better agreement (70%) than the hard filtering calls (61%) with the reference calls from Sanger sequencing. Overall, the comparisons against data from both GWAS and Sanger sequencing showed that VQSR provides better calling accuracy than simply using hard filtering. Thus, we used variant recalibration moving forward.

**Table 1:** UnifiedGenotyper Variant Quality Score Recalibration (UGVR) versus Hard Filter (UGHF)

		**UGHF**		
		**AA**	**AB**	**BB**
UGVR	AA	513,601	31 [5, 0, 0, 26]	49 [0, 0, 0, 49]
	AB	0	296,714	0
	BB	0	1,235 [0, 6, 1,222, 7]	170,818

Shown is a comparison of genotype calls from the two approaches for the 181,304 variants that were called by both and for which we had GWAS SNP genotypes. *A* refers to the reference allele and *B* to the alternative allele. The four values in brackets [*w, x, y, z*] refer to the genotype calls from the GWAS data, where *w* refers to homozygous reference (*AA*) calls, *x* to heterozygous (*AB*) calls, *y* homozygous alternative (*BB*) calls, and *z* to missing. The GWAS genotype calls are only shown for those calls that are discrepant between UGVR and UGHF. A total of 191,361 variants were called by both UGVR and UGHF. Of these, 181,304 (94.74%) were in common, 3,655 (1.91%) were unique to UGVR, and 6,402 (3.35%) were unique to UGHF.

### UnifiedGenotyper versus HaplotypeCaller

We next compared the accuracy of calls using the UnifiedGenotyper (UGVR) versus HaplotypeCaller (HCVR) algorithms as implemented in GATK version 2.5 (Table 2). Here, we used variant recalibration with both algorithms. Again, comparisons were made against GWAS genotype data from 100 subjects and 9,935 single nucleotide variations (SNVs). HaplotypeCaller calls variants via a local *de novo* assembly of haplotypes in an active region, while UnifiedGenotyper simply looks for a coincident haplotype event in the reads. Both methods evaluate haplotypes using an affine gap penalty Pair Hidden Markov Model [33]. However, UnifiedGenotyper uses a Bayesian genotype likelihood model and estimates the most likely genotype calls while HaplotypeCaller chooses the best two haplotypes which explain the read data [34]. Of the 190,352 SNVs called by either algorithm, 90.29% (171,867) were called in common. Among those SNVs called in common, the genotype calls were also highly concordant between the two algorithms (99.91%). Overall, the sensitivity and specificity of the calls from UnifiedGenotype versus HaplotypeCaller were nearly similar: 99.78% versus 99.80%, respectively, for sensitivity, and 99.68% versus 99.70%, respectively, for specificity. Among the few discordant genotype calls, the HCVR algorithm provided slightly more accurate calls than UGVR, when compared against the GWAS data. Of the 835 discordant genotype calls, the HCVR was correct 63.83% of the time as compared to 34.85% for UGVR. Both algorithms did equally well in calling homozygous alternative calls, but UGVR made a few more mistakes in making heterozygous calls when the true genotype was homozygous reference. Again, to evaluate the accuracy with respect to rarer SNVs (MAF <10%), we randomly selected 50 rarer SNVs from the subset that was discordantly called between UGVR and HCVR and performed Sanger sequencing to validate the call. The results were very similar to what we observed with comparisons against GWAS data. HCVR was correct 61% of the time as compared to 39% of the time for UGVR.

**Table 2:** UnifiedGenotyper Variant Quality Score Recalibration (UGVR) versus HaplotypeCaller Variant Quality Score Recalibration (HCVR)

		**UGVR**		
		**AA**	**AB**	**BB**
HCVR	AA	510,296	194 [176, 17, 0, 1]	[0, 0, 0, 0]
	AB	196 [60, 133, 2, 1]	294,595	210 [0, 5, 204, 1]
	BB	5 [0, 0, 5, 0]	230 [0, 10, 219, 1]	171,086

Shown is a comparison of genotype calls from the two approaches for the 465,681 variants that were called by both and for which we had GWAS SNP genotypes. *A* refers to the reference allele and *B* to the alternative allele. The four values in brackets [*w, x, y, z*] refer to the genotype calls from the GWAS data, where *w* refers to homozygous reference (*AA*) calls, *x* to heterozygous (*AB*) calls, *y* homozygous alternative (*BB*) calls, and *z* to missing. The GWAS genotype calls are only shown when the calls are discrepant between UGVR and HCVR. A total of 190,352 variants were called by both UGVR and UGHF. Of these, 171,867 (90.29%) were in common, 15,839 (8.32%) were unique to UGVR, and 2,646 (1.39%) were unique to HCVR.

#### Sequencing parameters

**Figure 4:**
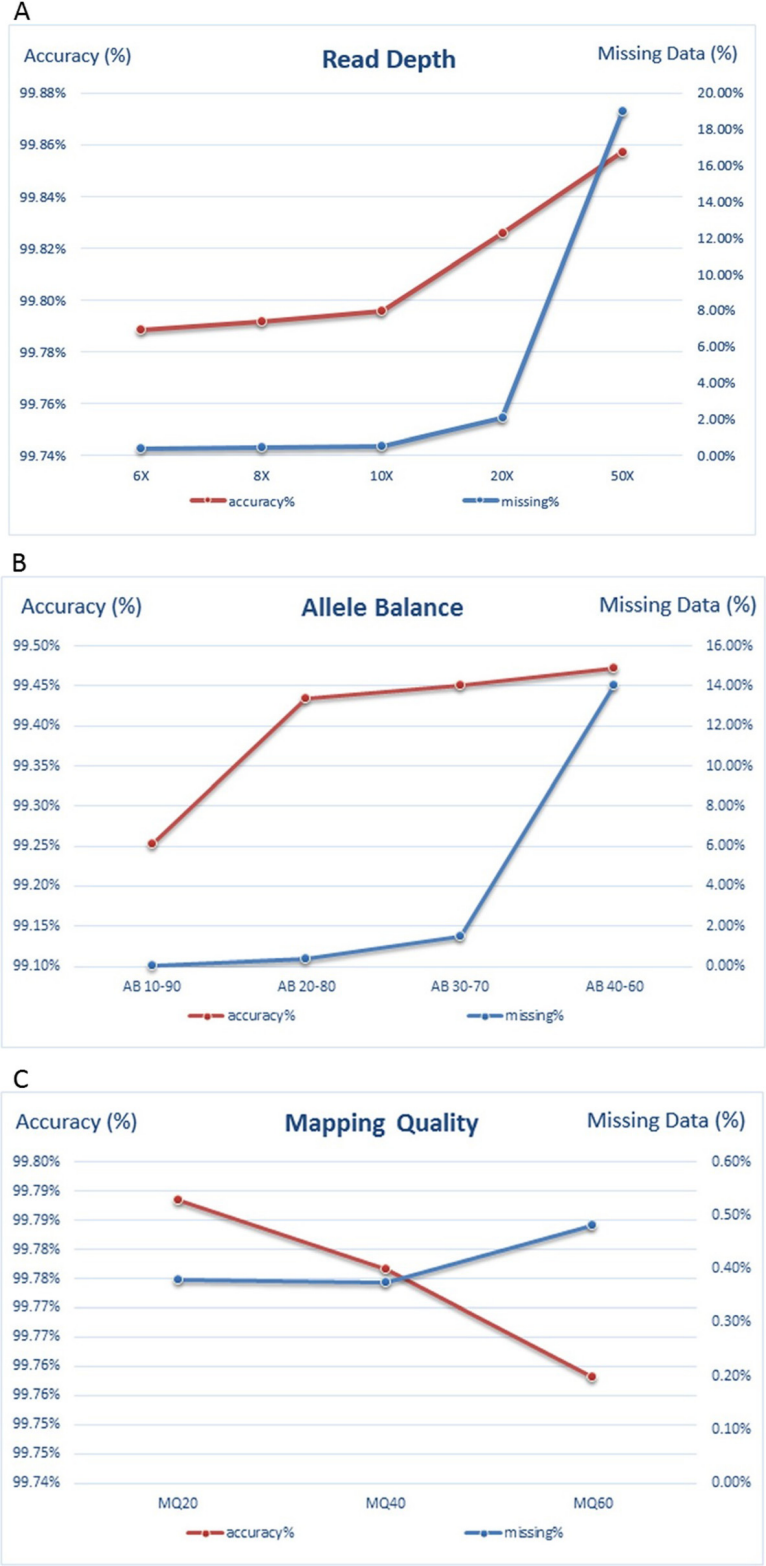
**Evaluation of the effect of sequencing parameters.** Read depth **(A)**, allele balance **(B)**, and mapping quality **(C)** on the calling accuracy. We compared the accuracy and missing data rates of the sequencing calls after systematically varying these parameters using data from 100 subjects with valid genotype data from GWAS on 7,370 SNPs.

Finally, we evaluated the effects of varying certain sequencing parameters such as read depth, allele balance, and mapping quality (Figure 4). We compared the accuracy and missing data rates of the sequencing calls after systematically varying these parameters using data from 100 subjects with valid genotype data from GWAS on 7,370 SNPs. Overall, the accuracy of the sequence calls, which were made using the UnifiedGenotyper algorithm and VQSR, was very high when compared with the GWAS genotype calls. However, several trends emerged. The accuracy of calls increased with both increasing read depth and allele balance towards 50-50. The increase in accuracy was most notable after read depths greater than 10 times, while it plateaued after allele balances between 20 and 80. The missing data rate similarly increased with read depth and allele balance as calls that did not meet the more stringent read depth or allele balance requirements were filtered. Thus, as expected, there was a trade-off between increasing accuracy and increasing missing data. This was not found for mapping quality. As mapping quality increased, the missing data rate also increased while the accuracy actually decreased. This might be explained by the fact that as the mapping quality criteria are increased, the number of reads that align to the reference genome decreases, leading to lower overall read depths on which to base downstream SNV calls and, as a result, lower accuracy calls. It is important to note, however, that these trends were relatively subtle and the overall accuracy of these calls made using best practices was well over 99%, regardless of the read depth, allele balance, and mapping quality thresholds.

## Conclusions

Advances in next-generation sequencing technologies have improved our ability to characterize genomic sequence variation at a scale and resolution not previously possible. This has opened up new avenues for studying how genetic variation contributes to human disease. A major challenge is how to process the copious data generated by the new technologies to yield high-quality data for downstream analyses. A variety of computational tools have been developed for this purpose. We have implemented a semi-automated pipeline using these tools to manage and analyze next-generation sequence data, and here we evaluated how key elements of the pipeline influence data quality.

After comparing SNV calls from GATK and SAMtools, we decided to adopt GATK [22] as our primary variant calling platform. In general, we found that GATK yields very high quality variant call data. Similar to others [23,31], we observed that realignment of mapped sequence reads around putative insertion/deletions (indels) and recalibration of base quality scores before variant calling are crucial to this performance. An example of the effects of realignment and recalibration on variant calling is illustrated in Additional file 3: Figure S1 and Additional file 4: Figure S2. For these comparisons between GATK and SAMtools with and without realignment/recalibration, we did not have SNP genotype data, and it was not practical to validate with Sanger sequencing non-calls by the different methods. As a result, we did not have information on false-negative and true-negative calls. Still, based on the available results from the validation of made calls, we felt confident in moving forward with GATK with realignment and recalibration.

GATK has developed several algorithms for variant calling from realigned and recalibrated sequence reads, including UnifiedGenotyper and HaplotypeCaller. Both performed well, but the HaplotypeCaller algorithm provided more accurate calls over all. Unlike UnifiedGenotype, HaplotypeCaller is capable of calling SNPs and insertion/deletion (indels) simultaneously. When the algorithm encounters a region that is highly variable, it discards the existing mapping information and reassembles the reads in the region *de novo*. The result is that HaplotypeCaller may be more accurate when calling regions that are traditionally difficult to call. This comes at a cost, however, as the HaplotypeCaller algorithm is currently computationally intensive, which limits the feasibility of using this approach with whole genome or larger exome sequencing studies. Improvements to the algorithm are needed to render it more efficient and practical to use with such studies. GATK has also implemented a Variant Quality Score Recalibration algorithm that uses machine learning methods for filtering variants that we demonstrated works better in terms of yielding a final set of accurate calls compared to hard filters based on pre-determined thresholds. Finally, we showed that there is a relationship between mapping quality, read depth and allele balance, and variant call accuracy, but if best practices are used throughout data processing, then additional filtering based on these metrics provides little gains.

Several previous studies have investigated factors that influence the accuracy of variant calling algorithms with sequence data [23-27]. One study sequenced 15 exomes from four families and processed the raw data using different alignment and variant-calling pipelines and found that there was a low concordance between approaches [25]. Another study used exome sequence data on 20 individuals and simulated whole genome sequence data to compare different algorithms for variant calling. Consistent with our results, this study found that GATK in particular outperformed SAMtools, especially for low coverage data, and yielded the most accurate data with multi-sample calling [27]. Still another study used whole genome sequence data from monozygotic twins to determine optimal sequencing filters for achieving the greatest concordance in variant calling at the minimal costs of filtered data [29]. However, similar to our study, work by the group that developed GATK suggested that variant recalibration with their machine learning approach performed better than strategies using hard filtering [25].

Our study has several strengths including having been carried out with real rather than simulated sequence data and having utilized direct comparisons against calls from more traditional platforms such as Sanger sequencing and GWAS microarray data that were previously validated. One limitation is that the comparisons against GWAS data were only for more common variants. It is unclear if the observed accuracy rates would be different for rarer variants that are not well represented in GWAS data. However, we note that when the sensitivity and specificity of SNV calls for lower frequency variants among the GWAS data (<20%) were examined, the results were not materially different from the more common variants (results not shown). In addition, we did not evaluate the quality of indel calls which pose their own challenges. Overall, the results reported here provide reassurance that it is possible to generate highly accurate data from next-generation sequencing. Our findings will help inform researchers who are seeking to optimize their own pipelines for working with next-generation sequence data. As tools and methods for processing such data are constantly evolving, we will continue to evaluate them to determine which can yield the highest-quality sequencing data.

## Methods

### Samples

Samples for the validation experiments described herein came from an ongoing whole exome sequencing study of bipolar disorder. A total of 130 samples were selected from two collections of pedigrees with bipolar disorder from Johns Hopkins or from the National Institute of Mental Health (NIMH) Genetics Initiative Bipolar Disorder Collaborative Study.

### Pre-capture library preparation

Genomic DNA samples were individually processed into Illumina paired-end or TruSeq DNA libraries using Illumina-compatible barcoded DNA adapters [17]. Purified genomic DNA, 1–3 μg, was initially fragmented using a Covaris S2 instrument (Covaris Inc, Woburn, MA, USA), followed by end-repair and ligation to paired-end adapters. As recommended by NimbleGen, pre-capture libraries were enriched with an additional 8 cycles of high-fidelity polymerase chain reaction (PCR). Pre-capture library quality and yield were assessed using the Bioanalyzer DNA 1000 Kit (Agilent Technologies, Santa Clara, CA, USA) and the NanoDrop 1000 Spectrophotometer (Thermo Scientific, West Palm Beach, FL, USA).

### Exome capture and sequencing

Due to ongoing changes in sequencing technology, sequencing was performed using two different exome capture kits and sequencing technologies. Our first set of analysis comparing SAMtools and GATK assessment was performed on sequencing data from 30 subjects captured with NimbleGen EZ exome v1.0 kit and sequenced with the Illumina Genome Analyzer (GA) II (Illumina Inc, San Diego, CA, USA). The NimbleGen EZ exome v1.0 kit was designed to capture approximately 33.8 Mb of hg18 genomic target, or approximately 180,000 coding exons from approximately 16,000 genes annotated in CCDS build 36.2 (April 2008 release). The remaining comparison analyses were carried out using 100 subjects that were captured with the NimbleGen EZ exome v2.0 kit and sequenced with the Illumina HiSeq 2000. The NimbleGen EZ exome v2.0 kit was designed to capture 36.0 Mb of hg19 genomic target, or approximately 300,000 coding exons from approximately 30,000 genes annotated across CCDS build 37.1 and RefSeq release 39. Sequencing generally produced enough coverage to obtain ≥80% of the target covered at ≥20X sequencing depth per sample. Samples that were just below this level (≥75% at 20X or more) were also included for further analyses. Variants were called using our pipeline as described in Additional file 1 and in more detail on our Wiki site (http://metamoodics.org/wes).

### Validation sequencing and genotyping

Next-generation sequence variant calls were validated against either Sanger sequencing or microarray genotyping from a previous GWAS. Sanger sequencing was carried out on a random selection of variants identified through our sequencing pipeline in 30 subjects. Multiple SNPs were assayed across all individual samples. SNPs were validated by Sanger sequencing. Primer pairs flanking each SNP were designed using Primer3 software (http://primer3.sourceforge.net/). Template DNA, 25–50 ng, was then used for amplification with the NEB LongAmp PCR protocol. Following amplification, PCR products were visualized on 1% agarose gels, and products which showed a single clean band in the proper size range were selected for further processing. PCR products were then incubated with exonuclease I to remove excess primers and shrimp alkaline phosphatase to remove unincorporated nucleotides. Sequencing reactions were performed using ABI BigDye terminator chemistry (Life Technologies, Austin, TX, USA). Reactions were then precipitated with salt and washed with ethanol. Samples were sequenced with both forward and reverse primers on the ABI 3730 sequencer. SNPs were confirmed using the CONSED software [35] to align the Sanger reads to the reference sequence and visualize the alleles at the putative SNP position. In the first validation round, 400 total SNPs were assayed. 330 of those were confidently genotyped, 21 were potentially genotyped but suffered from slightly messy data, and 49 failed due to poor data quality. In the second validation round, 300 total SNPs were assayed. Our of 248 that were confidently genotyped, 11 were potentially genotyped but suffered from slightly messy data, 37 failed due to poor data quality, and 4 were reported as a possible indel rather than a SNP.

In addition, for comparisons, we used SNP genotype data from a previously conducted GWAS. Details of the GWAS have been described elsewhere [36]. Briefly, samples were genotyped using the Affymetrix Genome-Wide Human SNP Array 6.0 (Affymetrix, Santa Clara, CA, USA) [37]. Allele calling was performed using the BirdSeed algorithm [38]. Scans from the same production plate were clustered together. Rigorous quality control measures were carried out with the resulting genotype calls. Samples were not used in the analysis if they had low call rate (<98.5%), excessively high (>0.363) or low (<0.344) heterozygosity, or incompatibility between reported gender and genetically determined gender [36]. Samples were also checked for unexpected familial relationships using pairwise IBD (Identity by Descent) estimation in PLINK [39]. SNPs were not analyzed if the minor allele frequency (MAF) was <0.01, the call rate was <95%, the SNP violated Hardy-Weinberg equilibrium (*p* < 1 × 10^−6^) in control samples within an ancestry group, there were ≥3 Mendelian errors, or there was >1 discrepancy among duplicate samples. Each plate in the study was compared to all other plates with a Chi-square test to examine and remove any plate effects.

## Abbreviations

BAM: binary alignment map; Indel: small insertion/deletion; NGS: next-generation sequencing; SAM: sequence alignment map; SNP: single-nucleotide polymorphism; SNV: single-nucleotide variant; VCF: variant call format; PLINK: population-based linkage analyses application.

## Competing interests

The authors declare that they have no competing interests. WRM has participated in Illumina-sponsored meetings over the past 4 years and received travel reimbursement and an honorarium for presenting at these events. Illumina had no role in decisions relating to the study/work to be published, data collection and analysis of data and the decision to publish. WRM has participated in Pacific Biosciences-sponsored meetings over the past 3 years and received travel reimbursement for presenting at these events. WRM is a founder and shared holder of Orion Genomics, which focuses on plant genomics and cancer genetics.

## Authors’ contributions

PPZ, JBP, and WDM conceived, designed, and directed the project. MP and MK designed the pipeline, coded, and ran the analysis. JP and MK performed the sequencing. JBP, WDM, PPZ, and FSG coordinated to the project. MP drafted the manuscript. All authors read, contributed to, and approved the final manuscript.

## Supplementary Material

Additional file 1 Whole Exome Sequencing Analysis Pipeline. Click here for file

Additional file 2: Table S1 Characteristics of the true-positive (TP) and false-positive (FP) variant calls for the comparisons of SAMtools without realignment/recalibrations, SAMtools with realignment/recalibration calls, and GATK with realignment/recalibration. Characteristics include functional annotation (using NCBI RefSeq, release 63), average read depth, number of variants in putative indels, and number of variants in repeat regions defined by UCSC simple tandem repeats track (hg19). Click here for file

Additional file 3: Figure S1 Illustration of SNVs at a specific locus using the integrated genomic viewer before (top) and after (bottom) applying realignment. Artefactual SNPs are recovered by realignment. Click here for file

Additional file 4: Figure S2 Illustration of changes in the quality scores and the residual errors by machine cycle before (left top and bottom) and after (right top and bottom) applying quality score recalibration. Click here for file
