# Supplementary material for: Validation and assessment of variant calling pipelines for next-generation sequencing
Source: Hum Genomics. 2014 Jul 30;8(1):14. doi: 10.1186/1479-7364-8-14 (PMC4129436; doi:10.1186/1479-7364-8-14)
Supplement: Additional file 1 — Whole Exome Sequencing Analysis Pipeline. [file 1479-7364-8-14-S1.pdf]

Whole Exome Sequencing Analysis Pipeline

Please visit our wiki site (http://metamoodics.org/wes) for the latest updates and access to the source code.

The pipeline contains the following steps:

- Mapping
- Filtering
- Realign/recalibration
- variant calling

Mapping

1. Mapping : Align short sequences to the human reference genome sequence database.

```
Align first batch
$bwa aln -l 30 -t $CPUs $hg19_ref $fastq_path1/$fastq_for_gz > $PWDS/fastq1.sai

Align second batch
$bwa aln -l 30 -t $CPUs $hg19_ref $fastq_path1/$fastq_rev_gz > $PWDS/fastq2.sai

Paired-end first batch
$bwa sampe  $hg19_ref $PWDS/fastq1.sai $PWDS/fastq2.sai $fastq_path1/$fastq_for_gz
$fastq_path1/$fastq_rev_gz | gzip > $PWDS/${subjectID}.sam.gz

Sorting bam
${samtools} view -uS $PWDS/${subjectID}.sam.gz  |  ${samtools} sort -m 3000000000 -
$PWDS/${subjectID}.srt

Indexing bam
${samtools} index ${PWDS}/${subjectID}.srt.bam

Generate stat
${bamtools} stats -insert -in $PWDS/${subjectID}.srt.bam > $PWDS/${subjectID}.srt.stats
```

2. Fixmate : Fixing the mate pairs information to ensure that all mate-pair information is in sync between each read and its mate pair.

```
 # Using Picard fixmate

java -Xmx${heap}m -Djava.io.tmpdir\=${tmp_folder}_fixmate \
 -jar ${picard}/FixMateInformation.jar \
 INPUT\=$PWDS/${subjectID}.srt.bam \
 OUTPUT\=$PWDS/${subjectID}.fxmt.bam \
 SO\=coordinate \
 CREATE_INDEX\=true  \
 VALIDATION_STRINGENCY\=SILENT
```

Filtering

1. Filter : Filtering for mapping, pairing, and proper paired

```
${bamtools} filter \
 -isMapped true \
 -isPaired true \
 -isProperPair true \
 -in  $PWDS/${subjectID}.fxmt.bam \
 -out $PWDS/${subjectID}.fxmt.flt.bam
```

2. Remove duplicate : Examines aligned records in the BAM file to locate duplicate reads and remove them.

```
java -Xmx${heap}m -Djava.io.tmpdir\=${tmp_folder}_rmdup \
 -jar ${picard}/MarkDuplicates.jar \
 I\=$PWDS/${subjectID}.fxmt.flt.bam \
 O\=$PWDS/${subjectID}.rmdup.bam \
 M\=$PWDS/${subjectID}.duplicate_report.txt \
 VALIDATION_STRINGENCY\=SILENT \
 REMOVE_DUPLICATES\=true
```

3. Filter low mapping quality : Filter low mapping quality reads

```
$bamtools   filter \
-mapQuality ">=60"    \
-in   $PWDS/${subjectID}.rmdup.bam   \
-out  $PWDS/${subjectID}.mq.srt.bam
```

Realign/Recalibration

1. Create intervals : Collect regions around potential indels and clusters of mismatches. Determine small suspicious intervals which are likely in need of realignment.

```
java -Xmx${heap}m -Djava.io.tmpdir\=${tmp_folder}_realign \
-jar $gatk \
-T RealignerTargetCreator \
-I $PWDS/${subjectID}.mq.srt.bam \
-R $REF \
-known $DBSNP  \
-nt $CPUs \
-o $PWDS/${subjectID}.forRealign.intervals   \
  -L $ExonFile
```

2. Realignment : Run the realigner over the intervals to create a cleaned version of the BAM file.

```
java -Xmx${heap}m -Djava.io.tmpdir\=${tmp_folder}_realign \
-jar $gatk \
-I $PWDS/${subjectID}.mq.srt.bam \
-R $REF \
-T IndelRealigner \
-targetIntervals $PWDS/${subjectID}.forRealign.intervals \
--out $PWDS/${subjectID}.realigned.bam \
-known $DBSNP  \
-LOD 0.4  \
-compress 5 \
-l INFO  \
  -L $ExonFile
```

3. Analysis of covariates : Determine the covariates affecting base quality scores in the BAM file.

```
 java -Xmx${heap}m -Djava.io.tmpdir\=${tmp_folder}_covar \
-jar $gatk \
-R $REF \
-l INFO \
-I $PWDS/${subjectID}.realigned.srt.bam \
-knownSites $DBSNP \
-T CountCovariates \
-nt $CPUs \
-cov ReadGroupCovariate \
-cov QualityScoreCovariate \
-cov CycleCovariate \
-cov DinucCovariate \
-recalFile $PWDS/${subjectID}.flt.recal_v1.csv  \
-L $ExonFile
```

4. Recalibration : Walking through the BAM file and rewrite the quality scores.

```
 java -Xmx${heap}m -Djava.io.tmpdir\=${tmp_folder}_recal \
-jar $gatk \
-l INFO \
-R $REF \
-I $PWDS/${subjectID}.realigned.srt.bam \
-T TableRecalibration \
--default_platform Illumina \
 --default_read_group MP1  \
--out $PWDS/${subjectID}.realigned.recal.bam \
-recalFile $PWDS/${subjectID}.flt.recal_v1.csv  \
-L $ExonFile
```

5. Recalculate analysis of covariates : Determine the covariates affecting base quality scores in the realigned recalibrated BAM file for the comparison.

```
 java -Xmx${heap}m -Djava.io.tmpdir\=${tmp_folder}_covar \
 -jar $gatk \
 -R $REF \
 -I $PWDS/${subjectID}.realigned.recal.bam \
 -knownSites ${DBSNP} \
 -T CountCovariates \
 -nt $CPUs \
 -cov ReadGroupCovariate \
 -cov QualityScoreCovariate \
 -cov CycleCovariate \
 -cov DinucCovariate \
 -recalFile $PWDS/${subjectID}.flt.recal_v2.csv  \
 -L $ExonFile
```

6. Depth of coverage : Determine coverage summarized by mean, median, quartiles, and/or percentage of bases covered.

```
 java -Xmx${heap}m -Djava.io.tmpdir\=${tmp_folder}_dept \
    -jar $gatk \
    -T DepthOfCoverage \
    -L $ExonFile \
    -l INFO \
    -R $REF \
    -I $PWDS/${subjectID}.realigned.recal.bam \
    -o $PWDS/${subjectID}.coverage.dept  \
    -L $ExonFile
```

7. DoC for genes : Determine coverage on genes

```
java -Xmx4g -jar GenomeAnalysisTK.jar    \
    -R  ucsc.hg19.fasta       \
    -T DepthOfCoverage    \
    -o   $i.depthofcoverage     \
    -I    $i.realigned.recal.bam         \
    -geneList   refGene.sorted.txt   \
    -ct 6 -ct 8 -ct 10 -ct 20   \
    -L   exon.target.interval_list \
    -omitBaseOutput \
    -omitLocusTable
```

8. HsMetrics : Calculates a set of Hybrid Selection specific metrics from an aligned BAM file.

```
java -Xmx${heap}m -jar ${picard}/CalculateHsMetrics.jar \
BAIT_INTERVALS\=$BaitFilePicard  \
TARGET_INTERVALS\=$ExonFilePicard   \
INPUT\=$PWDS/${subjectID}.realigned.recal.bam  \
OUTPUT\=$PWDS/${subjectID}.realigned.recal.hsmetrics
```

Variant calling

1. Calling variants

- Generate snps raw vcf file: Using GATK UnifiedGenotyper to generate snps.raw.vcf

```
java -Xmx${heap}m  \
   -jar $gatk \
   -R $REF \
   -T UnifiedGenotyper \
   --dbsnp $DBSNP \
   -I $PWDS/${subjectID}.realigned.recal.bam \
   --out $PWDS/variants/${subjectID}.snps.raw.vcf \
   -stand_call_conf 30.0 \
   -stand_emit_conf 10.0 \
   -out_mode EMIT_VARIANTS_ONLY \
   -l INFO \
   -A DepthOfCoverage \
   -A HaplotypeScore \
   -A InbreedingCoeff \
   -glm SNP  \
   -nt 1  \
   -L $ExonFile
```

- Generate indels raw vcf file: Using GATK UnifiedGenotyper to generate indels.raw.vcf

```
java -Xmx${heap}m  \
   -jar $gatk \
   -R $REF \
   -T UnifiedGenotyper \
   --dbsnp $DBSNP \
   -I $PWDS/${subjectID}.realigned.recal.bam \
   --out $PWDS/variants/${subjectID}.indels.raw.vcf \
   -stand_call_conf 30.0 \
   -stand_emit_conf 10.0 \
   -out_mode EMIT_VARIANTS_ONLY \
   -l INFO \
   -A DepthOfCoverage \
   -A HaplotypeScore \
   -A InbreedingCoeff \
   -glm INDEL  \
   -nt 1  \
   -L $ExonFile
```

- Variant filtration generate indels and indels.PASS files

```
java -Xmx${heap}m  \
 -jar $gatk \
 -l INFO -T VariantFiltration \
 -R $REF \
 -o $PWDS/variants/${subjectID}.indels.vcf \
 -V:VCF $PWDS/variants/${subjectID}.indels.raw.vcf \
 --filterExpression "QD < 2.0" \
 --filterName "QDFilter" \
 --filterExpression "ReadPosRankSum < -20.0" \
 --filterName "ReadPosFilter" \
 --filterExpression "FS > 200.0" \
 --filterName "FSFilter" \
 --filterExpression "MQ0 >= 4 && ((MQ0 / (1.0 * DP)) > 0.1)" \
 --filterName "HARD_TO_VALIDATE" \
 --filterExpression "QUAL < 30.0 || DP < 6 || DP > 5000 || HRun > 5" \
 --filterName "QualFilter"
```

- Variant filtration generate snps and snps.PASS files

```
java -Xmx${heap}m  \
 -jar $gatk \
 -l OFF -T VariantFiltration \
 -R $REF \
 -o $PWDS/variants/${subjectID}.snps.vcf \
 --variant $PWDS/variants/${subjectID}.snps.raw.vcf \
 --mask $PWDS/variants/${subjectID}.indels.PASS.vcf \
 --maskName InDel \
 --clusterSize 3 \
 --clusterWindowSize 10 \
 --filterExpression "QD < 2.0" \
 --filterName "QDFilter" \
 --filterExpression "MQ < 40.0" \
 --filterName "MQFilter" \
 --filterExpression "FS > 60.0" \
 --filterName "FSFilter" \
 --filterExpression "HaplotypeScore > 13.0" \
 --filterName "HaplotypeScoreFilter" \
 --filterExpression "MQRankSum < -12.5" \
 --filterName "MQRankSumFilter" \
 --filterExpression "ReadPosRankSum < -8.0" \
 --filterName "ReadPosRankSumFilter" \
 --filterExpression "QUAL < 30.0 || DP < 6 || DP > 5000 || HRun > 5" \
 --filterName "StandardFilters" \
 --filterExpression "MQ0 >= 4 && ((MQ0 / (1.0 * DP)) > 0.1)" \
 --filterName "HARD_TO_VALIDATE"
```

- evaluating snps: Using GATK snps eval to generate snps.PASS.vcf.eval file

```
java -Xmx${heap}m  \
  -jar $gatk \
  -T VariantEval \
  --dbsnp $DBSNP \
  -R $REF \
  -eval $PWDS/variants/${subjectID}.snps.PASS.vcf \
  -o $PWDS/variants/${subjectID}.snps.PASS.vcf.eval
```

2. Group Calling and Variant Recalibration

- UnifiedGenotyper Group Calling

```
 # SNPs

java -Xmx32g -Djava.io.tmpdir=/md1/BSI-SZ/analysis/tmp1 \
  -jar $GATK \
  -l INFO -L $targets_intervals  -nt 2  \
  -R $hg19_ref  \
 -I /md1/D12PEACXX_1_ACAGTG.realigned.recal.bam.g.bam \
 -I /md1/D12PEACXX_1_ACTTGA.realigned.recal.bam.g.bam \
 .
 . (list all bam files here)
 .
 -I /md1/D12PEACXX_8_TAGCTT.realigned.recal.bam.g.bam \
 -I /md1/D12PEACXX_8_TGACCA.realigned.recal.bam.g.bam \
 -I /md1/D12PEACXX_8_TTAGGC.realigned.recal.bam.g.bam \
  --dbsnp $dbsnp \
  -T UnifiedGenotyper  -glm $glmmode \
  -dcov 250 -out_mode EMIT_VARIANTS_ONLY \
  --max_alternate_alleles 10  \
  -o $outfile \
  -stand_call_conf 30 -stand_emit_conf 10

# Indels

java -Xmx32g -Djava.io.tmpdir=/md1/BSI-SZ/analysis/tmp2 \
  -jar $GATK \
  -l INFO -L $targets_intervals  -nt 8  \
  -R $hg19_ref  \
 -I /md1/D12PEACXX_1_ACAGTG.realigned.recal.bam.g.bam \
 -I /md1/D12PEACXX_1_ACTTGA.realigned.recal.bam.g.bam \
 -I /md1/D12PEACXX_1_ATCACG.realigned.recal.bam.g.bam \
 .
 . (list all bam files here)
 .
 -I /md1/D12PEACXX_8_TGACCA.realigned.recal.bam.g.bam \
 -I /md1/D12PEACXX_8_TTAGGC.realigned.recal.bam.g.bam \
  --dbsnp $dbsnp \
  -T UnifiedGenotyper  -glm $glmmode \
  -dcov 250 -out_mode EMIT_VARIANTS_ONLY \
  --max_alternate_alleles 10  \
  -o $outfile \
  -stand_call_conf 30 -stand_emit_conf 10
```

- <u>Variant Recalibration</u>

```
 # Variant Recalibrator for SNPs

java -Xmx32g -Djava.io.tmpdir=$wdir/tmp1                \
  -jar $GATK/GenomeAnalysisTK.jar                       \
  -l INFO -L $targets_intervals  -nt 4                  \
  -R $hg19_ref                                          \
  -T VariantRecalibrator                                \
  -input $wdir/$input_rawSNP                            \
  --maxGaussians 6                                      \
  -resource:hapmap,known=false,training=true,truth=true,prior=15.0   \
              $GATK/resources/hg19/hapmap_3.3.hg19.vcf          \
  -resource:omni,known=false,training=true,truth=false,prior=12.0     \
              $GATK/resources/hg19/1000G_omni2.5.hg19.vcf       \
  -resource:dbsnp,known=true,training=false,truth=false,prior=6.0     \
              $GATK/resources/hg19/dbsnp_137.hg19.vcf           \
  -an QD -an MQRankSum -an ReadPosRankSum -an FS -an MQ -an DP        \
  -an InbreedingCoeff -an HaplotypeScore                              \
  -mode SNP                                                           \
  -recalFile     $wdir/bsi.SNPrecal.vcf                               \
  -tranchesFile  $wdir/bsi.SNPrecal.tranches                          \
  -rscriptFile   $wdir/bsi.SNPrecal.plots.R




# Variant Recalibrator for Indels

java -Xmx32g -Djava.io.tmpdir=$wdir/tmp2                \
  -jar $GATK/GenomeAnalysisTK.jar                       \
  -l INFO -L $targets_intervals  -nt 4                  \
  -R $hg19_ref                                          \
  -T VariantRecalibrator                                \
  -input $wdir/$input_rawINDEL                          \
  --maxGaussians 1                                      \
  -percentBad 0.15 --minNumBadVariants 500              \
  -resource:mills,VCF,known=false,training=true,truth=true,prior=12.0     \
              $GATK/resources/hg19/Mills_and_1000G_gold_standard.indels.hg19.vcf     \
  -resource:dbsnp,known=true,training=false,truth=false,prior=2.0     \
              $GATK/resources/hg19/dbsnp_137.hg19.vcf       \
  -an DP -an FS -an ReadPosRankSum -an MQRankSum                 \
  -an QD -an ReadPosRankSum                                      \
  -an InbreedingCoeff                                            \
  -mode INDEL                                                    \
  -recalFile     $wdir/bsi.Indelrecal.vcf                        \
  -tranchesFile  $wdir/bsi.Indelrecal.tranches                   \
  -rscriptFile   $wdir/bsi.Indelrecal.plots.R
```

- Apply Recalibration

```
 # Apply Recalibration for SNPs

java -Xmx32g -Djava.io.tmpdir=$wdir/tmp1    \
  -jar $GATK/GenomeAnalysisTK.jar           \
  -l INFO -L $targets_intervals  -nt 4      \
  -R $hg19_ref                              \
  -T ApplyRecalibration                     \
  -input $wdir/$input_rawSNP                \
  --ts_filter_level 99.0                    \
  -recalFile    $wdir/bsi.SNPrecal.vcf      \
  -tranchesFile $wdir/bsi.SNPrecal.tranches \
  -mode SNP                                 \
  -o $wdir/bsi.VarRecalfiltSNP.vcf




# Apply Recalibration for Indels

java -Xmx32g -Djava.io.tmpdir=$wdir/tmp2          \
  -jar $GATK/GenomeAnalysisTK.jar                 \
  -l INFO -L $targets_intervals  -nt 4            \
  -R $hg19_ref                                    \
  -T ApplyRecalibration                           \
  -input $wdir/$input_rawINDEL                    \
  --ts_filter_level 95.0                          \
  -recalFile    $wdir/bsi.Indelrecal.vcf          \
  -tranchesFile $wdir/bsi.Indelrecal.tranches     \
  -mode INDEL                                     \
  -o $wdir/bsi.VarRecalfiltIndel.vcf
```

- Variant Evaluation

```
 # Variant Eval for SNPs

java -Xmx32g -Djava.io.tmpdir=$wdir/tmp1 \
  -jar $GATK/GenomeAnalysisTK.jar        \
  -l INFO -L $targets_intervals  -nt 4   \
  -R $hg19_ref                           \
  -T VariantEval                         \
  --dbsnp $dbsnp_ex                      \
  -eval  $wdir/bsi.VarRecalfiltSNP.vcf   \
  -o     $wdir/bsi.Final_SNP.gatkreport



# Variant Eval for Indels

java -Xmx32g -Djava.io.tmpdir=$wdir/tmp2   \
  -jar $GATK/GenomeAnalysisTK.jar          \
  -l INFO -L $targets_intervals  -nt 4     \
  -R $hg19_ref                             \
  -T VariantEval                           \
  --dbsnp $dbsnp_ex                        \
  -eval   $wdir/bsi.VarRecalfiltIndel.vcf  \
  -o      $wdir/bsi.Final_Indel.gatkreport
```

3. Annotation snpEff

```
java -Xmx${heap}m  \
  -jar   snpEff.jar  \
   eff   \
   -v -i vcf \
   -o vcf   \
   -c    ${snpEff}/snpEff.config
   GRCh37.65   \
   $PWDS/variants/${subjectID}.snps.PASS.vcf


java -Xmx${heap}m  \
  -jar   snpEff.jar  \
   eff   \
   -v -i vcf \
   -o vcf   \
   -c    ${snpEff}/snpEff.config
   GRCh37.65   \
   $PWDS/variants/${subjectID}.indels.PASS.vcf
```

4. Annotation Annovar

```
perl annotate_variation.pl --downdb --buildver hg19 refGene humandb
perl annotate_variation.pl --downdb --buildver hg19 knownGene humandb
perl annotate_variation.pl --downdb --buildver hg19 ensGene humandb
perl annotate_variation.pl --downdb --buildver hg19 1000g humandb/
perl annotate_variation.pl --downdb --buildver hg19 snp132 humandb/
perl annotate_variation.pl --downdb --buildver hg19 avsift humandb/
perl annotate_variation.pl --downdb --buildver hg19 1000g2012feb humandb/
perl annotate_variation.pl --downdb --buildver hg19  ljb_all --webfrom annovar  humandb/
annotate_variation.pl --downdb --buildver hg19  esp5400_ea -webfrom annovar humandb/
annotate_variation.pl --downdb --buildver hg19  esp5400_all -webfrom annovar humandb/
annotate_variation.pl --downdb --buildver hg19 genomicSuperDups humandb/
annotate_variation.pl --downdb --buildver hg19 phastConsElements46way humandb/

perl   /path/to/annovar/summarize_annovar.pl   --verdbsnp 135    --buildver hg19     \
   /path/to/humandb   input.bed   --outfile   output_sum
```
