# Supplementary material for: Validation and assessment of variant calling pipelines for next-generation sequencing
Source: Hum Genomics. 2014 Jul 30;8(1):14. doi: 10.1186/1479-7364-8-14 (PMC4129436; doi:10.1186/1479-7364-8-14)
Supplement: Additional file 2: Table S1 — Characteristics of the true-positive (TP) and false-positive (FP) variant calls for the comparisons of SAMtools without realignment/recalibrations, SAMtools with realignment/recalibration calls, and GATK with realignment/recalibration. Characteristics include functional annotation (using NCBI RefSeq, release 63), average read depth, number of variants in putative indels, and number of variants in repeat regions defined by UCSC simple tandem repeats track (hg19). [file 1479-7364-8-14-S2.docx]

Supplement Table 1: Characteristics of the true positive (TP) and false positive (FP) variant calls for the comparisons of SAMtools without realignment/recalibrations, SAMtools with realignment/recalibration calls, and GATK with realignment/recalibration. Characteristics include functional annotation (using NCBI RefSeq, release 63), average read depth, number of variants in putative indels, and number of variants in repeat regions defined by UCSC simple tandem repeats track (hg19).

|  | synonymous | nonsynonymous | read depth | Indel | repeat |
| --- | --- | --- | --- | --- | --- |
| **SAMtools without real/recal FP** | 8 | 4 | 57X | 52 | 4 |
| **SAMtools without real/recal TP** | 96 | 100 | 62X | - | 8 |
| **SAMtools with real/recal FP** | 2 | 4 | 34X | 4 | 1 |
| **SAMtools with real/recal TP** | 120 | 105 | 46X | - | 8 |
| **GATK with real/recal FP** | 3 | 4 | 35X | 5 | 1 |
| **GATK with real/recal TP** | 128 | 134 | 49X | - | 5 |
|  |  |  |  |  |  |
|  |  |  |  |  |  |
|  |  |  |  |  |  |
