# Supplementary figures and images for: Validation and assessment of variant calling pipelines for next-generation sequencing
Source: Hum Genomics. 2014 Jul 30;8(1):14. doi: 10.1186/1479-7364-8-14 (PMC4129436; doi:10.1186/1479-7364-8-14)

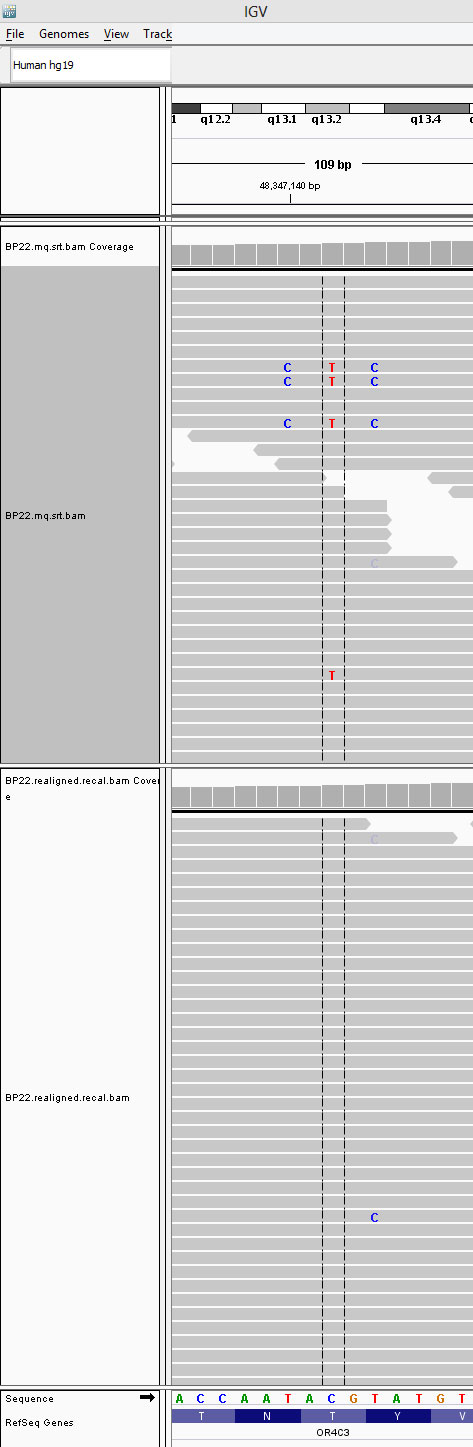

Supplement: Additional file 3: Figure S1 — Illustration of SNVs at a specific locus using the integrated genomic viewer before (top) and after (bottom) applying realignment. Artefactual SNPs are recovered by realignment. [file 1479-7364-8-14-S3.jpeg]

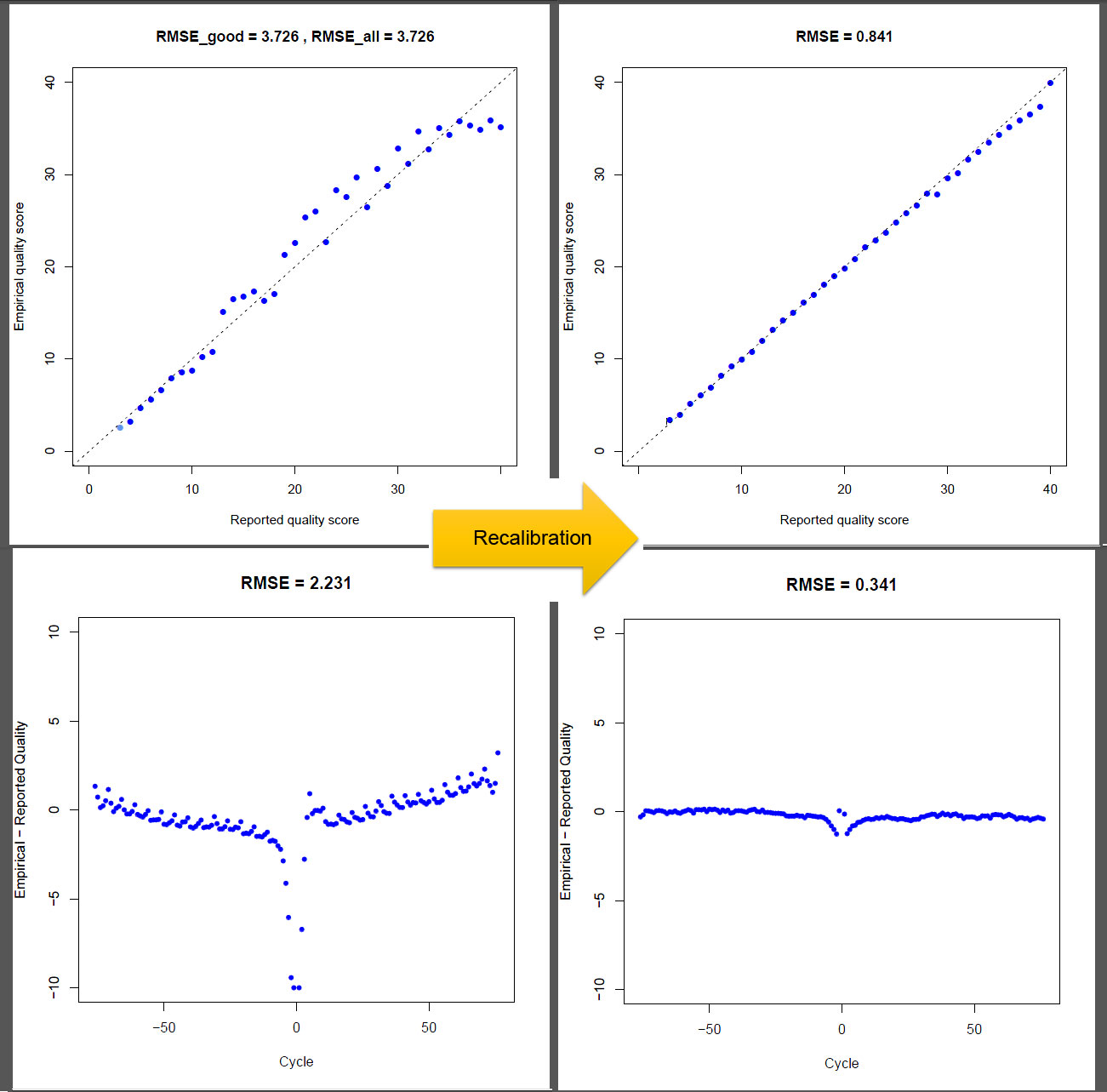

Supplement: Additional file 4: Figure S2 — Illustration of changes in the quality scores and the residual errors by machine cycle before (left top and bottom) and after (right top and bottom) applying quality score recalibration. [file 1479-7364-8-14-S4.jpeg]
